# Handbuch Der Vergleichenden Histologie Und Physiologie Der Haussangelthiere

**Published:** 1884-10

**Authors:** 


HANDBUCH DER VERGLEICHENDEN HISTOLOGIE UND PHYSIOLOGIE DER HAUSSAUGELTHIERE: Erster Band. Histologie der Haussäugelthiere für Thierarzte und Studirende, bearbeitet von Prof. Dr. Bonnet, et al., herausgegeben von Dr. W. Ellenberger, Erster Theil. Mit 204 gedruckten Holzschnitten in der Text. Berlin: Verlag von Paul Parey, 1884. M. 12, 8vo, pp, 308.

